# Inhibition of the lectin pathway of complement activation reduces LPS-induced acute respiratory distress syndrome in mice

**DOI:** 10.3389/fimmu.2023.1192767

**Published:** 2023-05-30

**Authors:** Youssif M. Ali, Nicholas J. Lynch, Ahmed A. Shaaban, Dina E. Rizk, Shaymaa H. Abdel-Rahman, Priyanka Khatri, Munehisa Yabuki, Sadam Yaseen, Thomas Dudler, Gregory Demopulos, Wilhelm J. Schwaeble

**Affiliations:** ^1^ Department of Veterinary Medicine, School of Biological Sciences, University of Cambridge, Cambridge, United Kingdom; ^2^ Department of Microbiology and Immunology, Faculty of Pharmacy, Mansoura University, Mansoura, Egypt; ^3^ Department of Pharmacology and Toxicology, Faculty of Pharmacy, Mansoura University, Mansoura, Egypt; ^4^ Omeros Corporation, Seattle, WA, United States

**Keywords:** ARDS, lectin pathway, lung injury, complement therapeutics, LPS

## Abstract

Acute respiratory distress syndrome (ARDS) is a life-threatening disorder with a high rate of mortality. Complement activation in ARDS initiates a robust inflammatory reaction that can cause progressive endothelial injury in the lung. Here, we tested whether inhibition of the lectin pathway of complement could reduce the pathology and improve the outcomes in a murine model of LPS-induced lung injury that closely mimics ARDS in human. *In vitro*, LPS binds to murine and human collectin 11, human MBL and murine MBL-A, but not to C1q, the recognition subcomponent of the classical pathway. This binding initiates deposition of the complement activation products C3b, C4b and C5b-9 on LPS *via* the lectin pathway. HG-4, a monoclonal antibody that targets MASP-2, a key enzyme in the lectin pathway, inhibited lectin pathway functional activity *in vitro*, with an IC_50_ of circa 10nM. Administration of HG4 (5mg/kg) in mice led to almost complete inhibition of the lectin pathway activation for 48hrs, and 50% inhibition at 60hrs post administration. Inhibition of the lectin pathway in mice prior to LPS-induced lung injury improved all pathological markers tested. HG4 reduces the protein concentration in bronchoalveolar lavage fluid (p<0.0001) and levels of myeloid peroxide (p<0.0001), LDH (p<0.0001), TNFα and IL6 (both p<0.0001). Lung injury was significantly reduced (p<0.001) and the survival time of the mice increased (p<0.01). From the previous findings we concluded that inhibition of the lectin pathway has the potential to prevent ARDS pathology.

## Introduction

Acute respiratory distress syndrome (ARDS) is a severe inflammation of the lung endothelial and epithelial barriers and is a major complication of acute lung injury in critical care medicine. Several factors can lead to ARDS such as aspiration, severe lung infection, or lung injury caused by inhalation of irritant gases. Extra pulmonary diseases such as sepsis or pancreatitis can also cause ARDS (1).

Lipopolysaccharide (LPS), the main glycolipid component of the outer membrane of Gram-negative bacteria, plays a key role in host–pathogen interactions with the innate immune system. LPS is known for its potent activation of many inflammatory cells, such as monocytes and macrophages, and triggers the release of a vast number of inflammatory cytokines (2). This inflammatory cascade is initiated when immunocytes recognise and bind with high affinity to LPS through the Toll-like receptor 4 resulting in the production of pro-inflammatory cytokines, which cause a robust inflammatory response (3).

The complement cascade is an integral part of the innate immune system and co-ordinates the interaction between the innate and adaptive immune systems (4, 5). Complement is activated *via* three pathways: the classical pathway (CP), the lectin pathway (LP) and the alternative pathway (AP). The CP is initiated through the binding of C1q to immune complexes, followed by the stepwise activation of two associated zymogens, C1r and C1s. The LP is initiated by binding of recognition molecules to carbohydrate motifs on the surface of microbes or to aberrant glycosylation or acetylation patterns on apoptotic, necrotic, malignant, or damaged host cells, followed by the activation of MASP-2 (the effector enzyme of the LP). MASP-1 has a supportive role in the activation of the lectin pathway but cannot compensate for the loss of MASP-2 functional activity as MASP-2 is the only lectin pathway associated enzyme that can cleave C4 at a physiologically meaningful rate (6).

Activation of either the CP or the LP pathway leads to cleavage of C4 and C2 followed by the formation of a C3 convertase (C4b2a), which splits C3 into the biologically active fragments C3b and C3a (6). The LP recognition subcomponents in humans are: the three collectins (mannan-binding lectin [MBL], CL-10, and CL-11), and three different ficolins (ficolins 1, 2, and 3) (6). Unlike humans, rodents express 4 LP-activating collectins (i.e., two forms of MBL known as MBL-A and MBL-C as well as CL-10 and CL-11) and two different forms of ficolins (ficolin A and ficolin B) (7).

Turnover of the AP is initiated either by the provision of C3b *via* the CP or LP pathway, or *via* the spontaneous hydrolysis of C3 to form C3(H2O) that binds to factor B, which is then cleaved by factor D to form the AP C3 convertase C3bBb or C3(H_2_O)Bb. Binding of C3b to C3 convertases (C4b2a or C3bBb) forms the C5 convertases, C4b2a(C3b)n or C3bBb(C3b)n, that cleave C5 into C5b and C5a. C5b binds with C6, C7, C8, and C9 to form the terminal pathway membrane attack complex (8). When complement is activated on target cell surfaces, the terminal complement components (C5b through C8 as well as up to nine or more C9 molecules) form the membrane attack complex (MAC). Insertion of MAC into the target cell results in the formation of pores, causing leakage of cytoplasmic contents, and the direct killing of Gram-negative bacteria. This process can also lyse erythrocytes or cause MAC deposition on other eukaryotic cells (9).

LPS from diverse Gram-negative bacteria initiates the complement system *via* different pathways. Complement activation fragments C3a and C5a are anaphylatoxins, potent inflammatory mediators that recruit monocytes and neutrophils into lung tissue, initiating a severe inflammatory reaction (10, 11). Massive infiltration of neutrophils into the lung tissues, and activation of resident alveolar macrophages that secrete a wide range of inflammatory cytokines as well as the release of nitric oxide and reactive oxygen species, initiates a second wave of inflammatory stimulators, such as platelet activating factors and leukotrienes that cause intrapulmonary haemorrhage and severe lung injury, ending in ARDS and, finally, tissue necrosis and lung damage (12–15).

Although ARDS is a life-threatening complication, the therapeutic interventions available for treatment are limited; patients can be placed in the prone position, given sedatives to reduce movement, or placed on mechanical ventilation or extracorporeal membrane oxygenation (ECMO) (16).

During the COVID-19 pandemic, several clinical trials were run to assess complement inhibitors as a novel therapeutic approach for the treatment of ARDS associated with severe cases of COVID-19. Treatment of severe acute COVID-19 patients with narsoplimab, a fully humanised monoclonal antibody targeting MASP-2, resulted in improved clinical status and laboratory findings, and showed the largest reduction in mortality risk across all drugs reported from the I-SPY COVID-19 platform trial (17). Narsoplimab has also shown clinical benefit across multiple other indications, including hematopoietic stem cell transplant-associated thrombotic microangiopathy and immunoglobulin A nephropathy (18, 19). In this study we assessed to what extent LP inhibition, using a monoclonal antibody derivative of narsoplimab that specifically inhibits MASP-2, reduces the LPS-mediated lung injury in an established mouse model of ARDS.

## Materials and methods

### Ethics statement

Animal procedures were approved by the local Ethical Committee of the Faculty of Pharmacy, Mansoura University, Egypt, and Animal Welfare and Ethical Review Body (AWERB), University of Cambridge under the Home Office Project Licence numbers PP273509.

### Binding ELISA assays

Microtiter ELISA plates (NUNC, Maxisorp) were coated with 100μL of 2 μg/mL of LPS in carbonate buffer or control substrates including: 10μg/mL mannan, 10μg/mL zymosan, 10μg/mL N-acetylated BSA or an immune complex prepared by incubating bovine serum albumin (BSA) with anti-BSA. After overnight incubation at 4°C, ELISA plates were blocked using 2% BSA in TBS for 2h at room temperature. Plates were then washed with TBS containing 0.05% (v/v) Tween 20 and 5mM CaCl_2_. Normal human serum (NHS) or wild-type (WT) mouse serum were diluted in BBS (4mM barbital, 14 mM NaCl, 2mM CaCl_2_, 1mM MgCl_2_, pH 7.4) and incubated with the plates for 1h at 37°C. Plates were washed and binding of recognition molecules was detected using goat anti-CL-11(Santa-Cruz Biotechnology), rabbit anti-human L-ficolin, mouse anti-human CL-11, rat anti-mouse MBL-A, rat anti-mouse MBL-C, rabbit anti-mouse ficolin-A or rabbit anti-C1q antibodies. Bound antibodies were quantified using HRP-conjugate secondary antibodies and the chromogenic substrate TMB (Sigma) (20).

### Complement activation assay

ELISA plates were coated with 10μg/mL mannan or 10μg/mL LPS then blocked as described above. WT mouse serum or NHS were diluted in BBS buffer, added to the plate and incubated for 1h at 37°C. Binding of C3b or C4b were detected using either rabbit anti-C3c (Dako) or rabbit anti-C4c (Dako), mouse anti-human C5b-9 (Dako) or rat anti mouse C5b-9, respectively, followed by HRP conjugated goat anti-rabbit IgG, rabbit anti-human or rabbit anti-rat antibodies. To assess complement C3b deposition *via* the AP, serum samples were diluted in EGTA buffer (4 mM barbital, 145 mM NaCl, 20mM EGTA, 2mM MgCl_2_, pH 7.4), a buffer that prevents C3b deposition *via* the CP or LP while leaving the AP unaffected.

### Evaluation of HG4 functional activity

The inhibitory anti-MASP-2 antibody (HG4) used in this study is an IgG4 derivative of the human MASP-2 inhibitory monoclonal antibody narsoplimab (OMS721) modified for improved LP inhibition in mice (21). To assess the efficacy of the antibody *in vitro*, 50% mouse serum in the presence of different concentrations of HG4 was exposed to an ELISA plate previously coated with mannan or an immune complex. Complement C3b or C4b deposition was measured as previously described (21). To evaluate the inhibitory effect of HG4 *in vivo*, a group of male BALB/c mice were injected intraperitoneally with either 5mg/kg of HG4 or an isotype control antibody. Serum samples were collected at different time points and the levels of LP inhibition were evaluated for residual LP-mediated C4b deposition as described above.

### LPS-induced lung injury in mice

12-week-old male BALB/c mice were used in this study. Mice were administered intraperitoneally (i.p.) either 5mg/kg of monoclonal antibody HG4 or an isotype control antibody. After 12h, mice were lightly anaesthetized using 2.5% (v/v) fluothane (AstraZeneca, Macclesfield, UK) over oxygen (1.5 to 2 l/min). Each mouse received 150μg LPS (Escherichia coli, serotype O111:B4; Sigma-Aldrich) in 50μL phosphate buffered saline (PBS) *via* intranasal route. Control mice received only PBS. Animals were monitored for signs and symptoms of disease progression.

### Collections of Bronchoalveolar lavage fluid (BALF) for analysis

Mice (n=5) were euthanised at predetermined time points (24hrs and 48hrs) and lungs were lavaged three times with one mL PBS. Bronchoalveolar lavage fluid (BALF) was centrifuged at 800×g and the supernatant was isolated and kept frozen for further analysis. Cell pellets were re-suspended in 0.5mL PBS for differential count using haemocytometer as previously described (22).

### Histopathology

Mice were euthanised at pre-determined time points and then lungs (n=3) were collected and fixed using 4%paraformaldehyde in PBS. Lung sections (5 μm) were stained with haematoxylin and eosin and subsequently fixed with DPX mount (BDH). Lung injury and pathology score were assessed by three independent researchers as described elsewhere (23).

### Cytokines and LDH levels analysis

The activity of the inflammatory mediators in BALF were measured using commercially available ELISA kits (R&D systems, UK) according to the manufacturer’s protocol. The levels of LDH in lung tissue homogenate was measured using lactate dehydrogenase (LDH) activity colorimetric assay kit.

### Measurement of myeloperoxidase activity in lung tissues

Lung tissues were homogenised in 50mM potassium phosphate buffer (pH7.4) containing 0.5% hexadecyl trimethyl ammonium bromide supplemented with 5 mmol/L EDTA. The supernatant fluid was collected after centrifugation at 12,000xg at 4 °C and then incubated with 50 mM potassium phosphate buffer containing the substrate, 1.5 mM H_2_O_2_ and 167 μg/ml O-dianisidine dihydrochloride (Sigma-Aldrich). The enzymatic activity was determined by measuring the absorbance at 460 nm over 3 minutes.

## Results

### LPS binds to LP recognition molecules, but not to C1q

**Figure 1:**
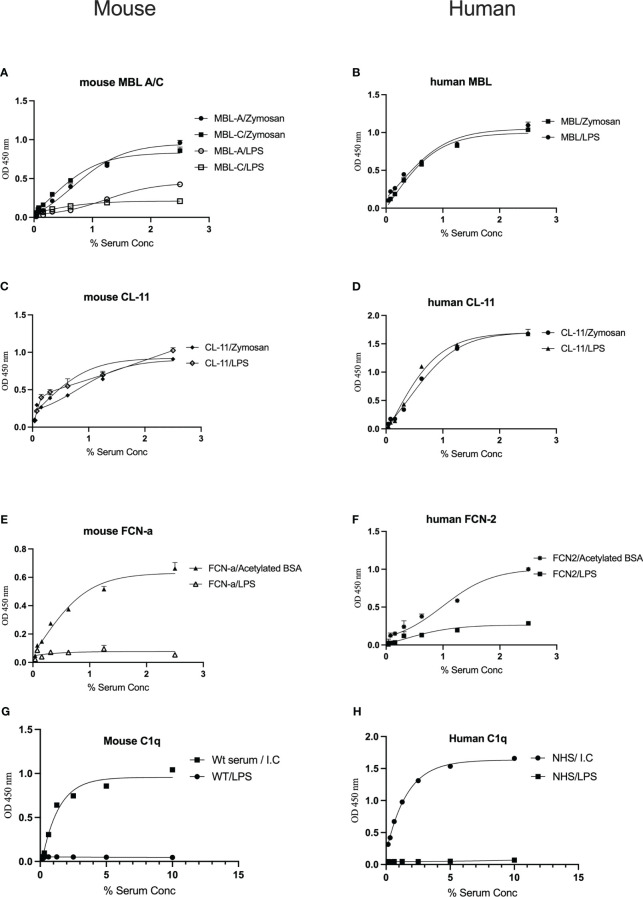
Binding of the LP recognition molecules and C1q to LPS. Solid-phase binding assays were used to assess the binding of the LP recognition molecules from murine **(A, C, E)** or human sera **(B, D, F)** to LPS. High levels of CL-11, mouse MBL-C and human MBL were observed. Neither ficolins nor C1q bound to LPS **(G, H)**. Results are means of duplicates ± SD.

To determine whether complement recognition molecules from human or murine serum bind to LPS, ELISA plates coated with LPS or control substrates were incubated with WT mouse serum or NHS, followed by evaluation of lectin binding. Murine MBL-A binds to LPS, whereas MBL-C (**Figure 1A**) and ficolin-A (Fcn-a) (**Figure 1E**) do not. In humans, MBL but not L-ficolin showed significant binding to LPS (**Figures 1B, F**). Both murine and human CL-11 bound to LPS in a concentration-dependent and saturable manner (**Figures 1C, D**). The CP recognition component C1q did not bind to LPS (**Figures 1G, H**).

### LPS induces LP-mediated complement activation

**Figure 2:**
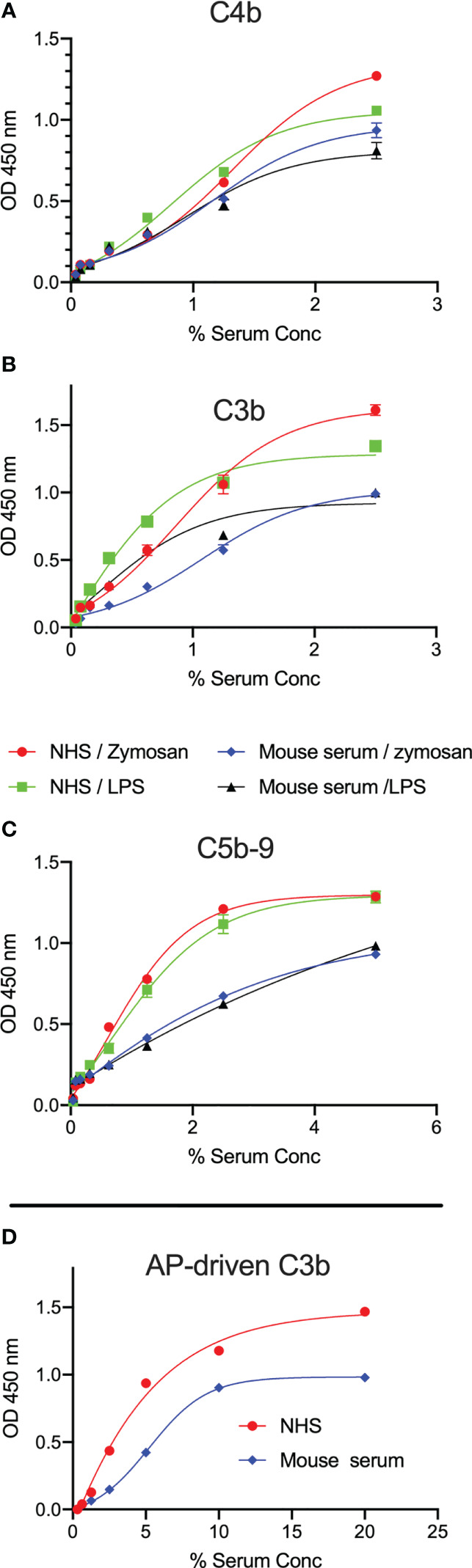
LPS promotes C3b, C4b and C5b-9 deposition from mouse and human sera. ELISA plates coated with zymosan or LPS were incubated with either wild-type mouse serum (Wt) or NHS for 1h at 37°C. High levels of C3b **(A)**, C4b **(B)** and C5b-9 **(C)** deposition in both mouse and human sera were detected on the surface of LPS at low serum concentrations. In a separate experiment, C3b deposition mediated via the alternative pathway (AP) was detected on LPS **(D)**. Results are means of duplicates ± SD.

Activation of complement was evaluated by measuring the levels of C3b, C4b and C5b-9 deposition on microtiter plates coated with LPS. High levels of complement C3b, C4b and C5b-9 deposition on LPS were observed using either mouse or human sera as a source of complement (**Figures 2A–C**). Since our data showed no binding of C1q to the surface of LPS, C3b and C4b deposition is likely due to LP activation. A robust C3b deposition mediated via the AP was also detected (**Figure 2D**).

### HG4 inhibits LP-mediated C4b deposition in mice

**Figure 3:**
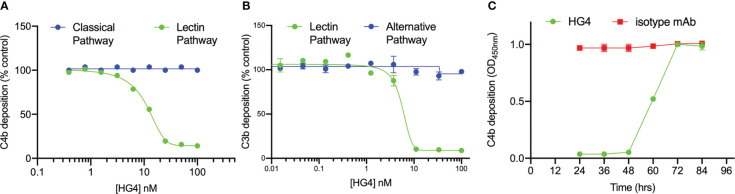
HG4 specifically inhibits LP in mice with no effect on CP or AP functional activity. Different concentrations of HG4 were mixed with 50% wild-type mouse serum in BBS buffer then incubated with an ELISA plate coated with either zymosan or an immune complex. C4b deposition was detected as described in the materials and methods. HG4 inhibits LP-mediated C4b deposition on mannan while it has no effect on CP-mediated C4b deposition on the immune complex **(A)**. In a parallel experiment, 50% mouse serum was diluted in BBS or EGTA buffer (EGTA buffer allows only C3b deposition *via* the AP) and then incubated with an ELISA plate coated with zymosan. C3b deposition was detected using anti-C3c antibodies. HG4 inhibits C3b deposition *via* the LP and has no effect on AP-mediated C3b deposition **(B)**. To assess the ability of HG4 to inhibit the LP *in vivo*, mice were treated with either HG4 or an isotype control and serum samples were collected at different time points. LP-mediated C4b deposition was assessed. In mice treated with HG4, LP activity was undetectable for 48 hr, followed by a gradual return over time, while no change in LP activity was seen in mice that received an isotype control antibody **(C)**.

The ability of HG4 to inhibit LP functional activity *in vitro* was tested by incubating serial dilutions of HG4 with 50% mouse serum in microtiter plates coated with mannan or an immune complex (to evaluate the effect on the CP) and subsequently determining the amount of C4b deposited on the plates. HG4 inhibited C4b deposition on mannan at a very low concentration, with an IC_50_ value of approximately 10nM (**Figure 3A**), demonstrating effective blockade of mouse LP activity with no inhibitory effect on CP functional activity. To evaluate if HG4 has an inhibitory effect on AP functional activity, complement C3b deposition *via* the AP was measured in the presence of different concentrations of HG4. AP functional activity remained intact in the presence of high concentrations of HG4 (**Figure 3B**). Inhibition of the LP *in vivo* was evaluated by injecting mice intraperitoneally with either 5mg/kg of HG4 or an isotype control antibody. Serum samples were collected at different time points and assessed for residual LP activity. LP-mediated C4b deposition on mannan was completely inhibited for 60h in sera collected from mice that received HG4, while no inhibition was seen in mice given an isotype control antibody (**Figure 3C**).

### Inhibition of the LP prevents the breakdown of the alveolar-capillary barrier in a murine model of lung shock injury

**Figure 4:**
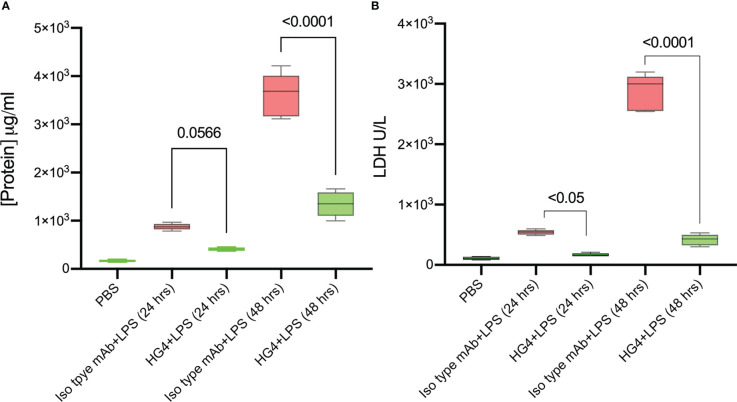
HG4 reduces the protein concentration and LDH levels in mice challenged with LPS. Mice were treated intraperitoneally with either 5mg/kg of HG4 or a control mAb 12 h before LPS administration. Mice treated with HG4 showed a significantly lower protein concentration in BALF **(A)** and reduced levels of LDH in lung tissues homogenate **(B)** compared to mice treated with LPS alone. BALF from mice that received PBS instead of LPS was used as a control. (n = 5). Results were analysed by ANOVA, with Tukey’s test for multiple comparisons.

One of the defining features of ARDS is severe inflammation, leading to increased vascular permeability, breakdown of the alveolar-capillary barrier, pulmonary oedema, and intrapulmonary haemorrhage. Having shown that the LP is instrumental in driving complement activation on LPS, we tested whether inhibition of the LP would ameliorate injury in a murine model of LPS-induced lung shock injury. The chosen LP inhibitor was HG4, a MASP-2-specific monoclonal antibody selected for effective inhibition of MASP-2 functional activity in mice. Alveolar–capillary barrier permeability was evaluated by measuring the protein content in BALF. Mice that received HG4 showed a significantly lower protein content in BALF compared to mice treated with an isotype control antibody 1 and 2 days after LPS administration (**Figure 4A**). The level of LDH, a biological marker for tissue and vascular damage during lung injury was also evaluated. HG4 significantly reduces the levels of LHD in mice treated with LPS compared to the group received an isotype control antibody (**Figure 4B**).

### Administration of HG4 significantly reduces alveolar inflammation in mice after LPS administration

**Figure 5:**
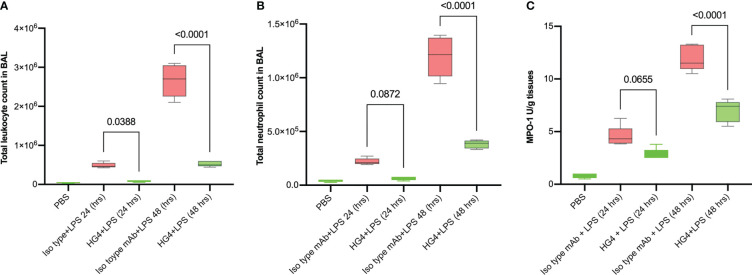
HG4 reduces inflammatory cell infiltration in the BALF of mice administered LPS. Mice were treated intraperitoneally either with 5mg/kg of HG4 or control mAb 12 h before LPS administration. Mice treated with HG4 showed a significantly lower total leukocyte count **(A)** and significantly fewer PMNs **(B)** in BALF when compared to mice treated with LPS alone. The levels of MPO in lung homogenate were significantly reduced in mice treated with HG4 compared to mice that received the isotype control antibody **(C)**. BALF from mice that received PBS instead of LPS was used as a control. Results were analysed by ANOVA, with Tukey’s test for multiple comparisons.

In mice challenged with LPS, treatment with a LP inhibitor HG4 significantly reduces infiltration of neutrophils into lungs compared to mice treated with an isotype control antibody. BALF cytology was dramatically changed in cellularity within the alveolar space during LPS-induced lung injury. The total number of leukocytes (especially neutrophils) was significantly greater in BALF of mice treated with the isotype control antibody compared to mice that received HG4 antibody treatment. The leukocyte count peaked 48h after LPS administration (**Figures 5A, B**).

The level of myeloperoxidase (MPO) activity in lung tissues was also measured. MPO is an enzyme stored in azurophilic granules of polymorphonuclear neutrophils (PMNs) and macrophages. During the inflammatory process, high levels of MPO are released into the local inflammatory environment. MPO activity was significantly reduced in mice treated with HG4 compared to mice treated with an isotype control antibody (**Figure 5C**).

**Figure 6:**
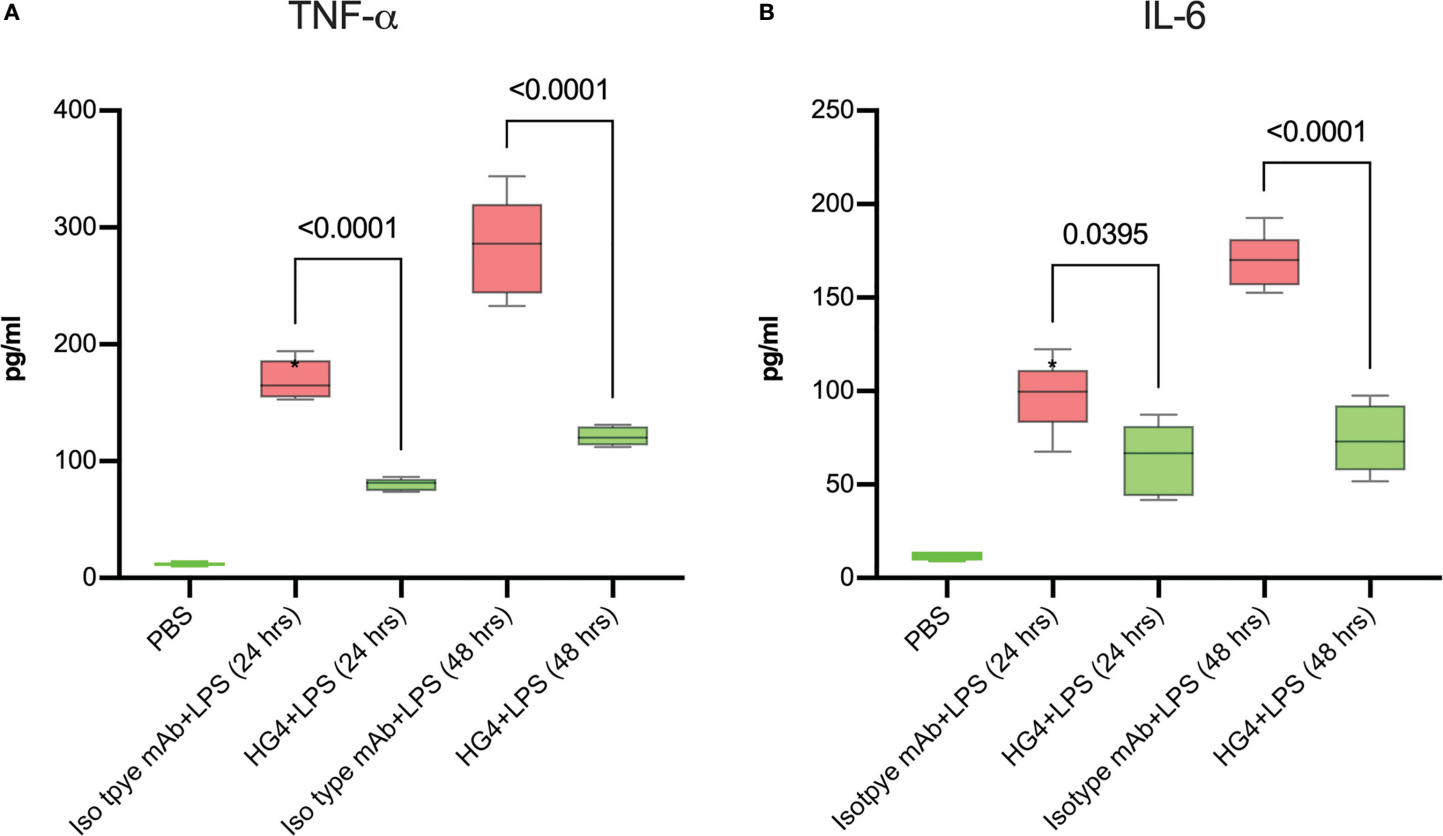
HG4 reduces the levels of pro-inflammatory cytokines in lungs of mice after LPS administration. The levels of TNF-α and IL-6 in BALF of mice were measured using an ELISA assay. Administration of HG4 significantly reduces TNF-a **(A)** and IL-6 **(B)** levels in BALF of mice after LPS administration compared to mice treated with isotype control antibodies. N=5 per group, results were analysed by ANOVA, with Tukey’s test for multiple comparisons.

In addition, HG4-treated animals showed a remarkable decrease in the levels of pro-inflammatory cytokines (IL1-β, IL-6 and TNF-α) in lung BALF after LPS administration compared to mice treated with isotype control antibodies (**Figure 6**).

### HG4 pre-treatment protects from lung injury and mortality associated with LPS administration

**Figure 7:**
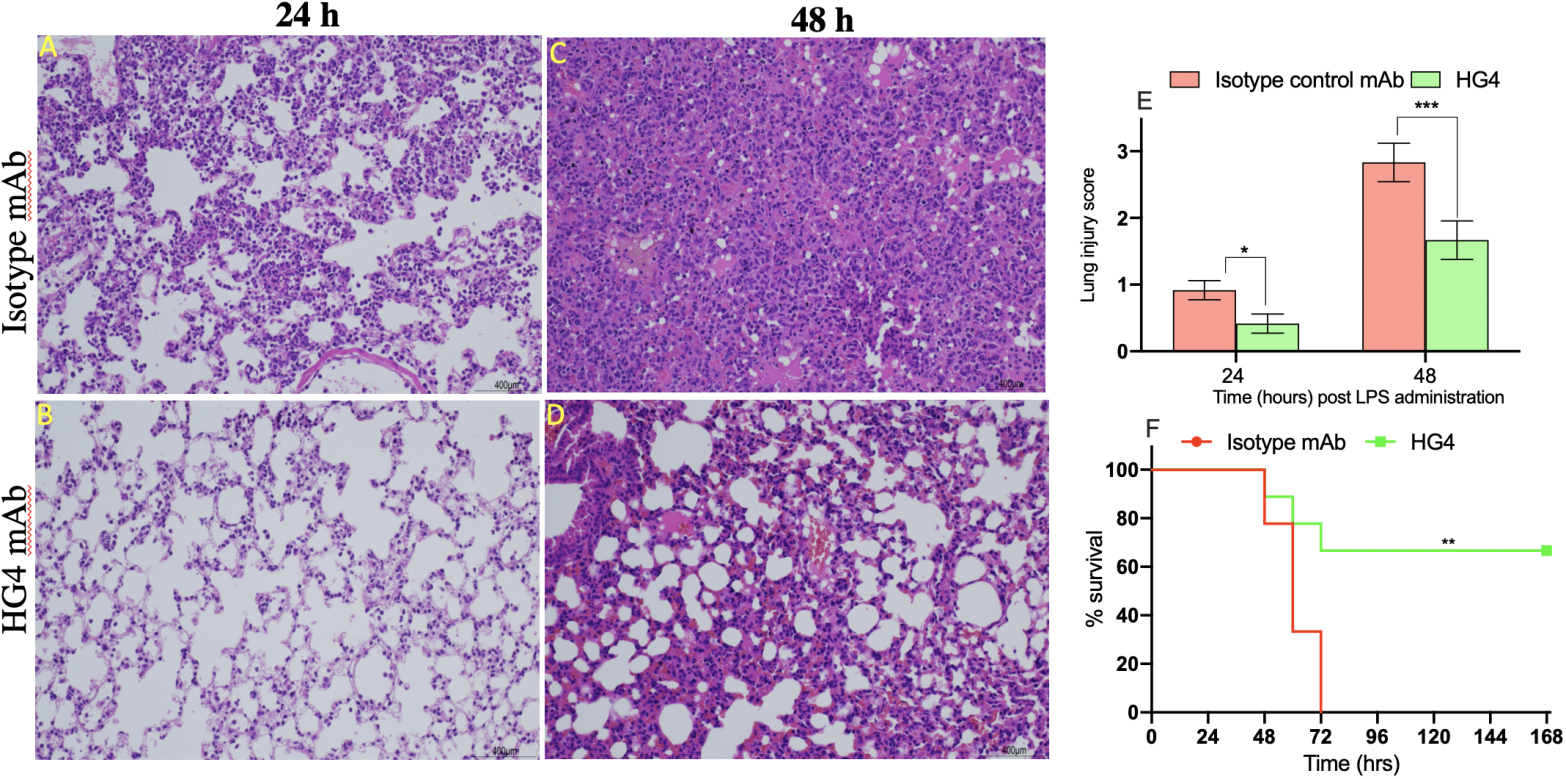
Therapeutic application of HG4 significantly reduced lung pathology after intranasal administration of LPS. Lung sections were taken at different time points after LPS administration and stained with H&E. Sections from animals treated with HG4 **(B, D)** showed a marked improvement of the lung pathology, with a significant reduction in leukocyte infiltration into the lung parenchyma and a pronounced reduction in perivascular oedema and mucous secretion, compared to lung sections from mice treated with the isotype control antibody **(A, C)** at days 1 and 2 post LPS administration. There was significantly lesser lung tissue damage in mice treated with HG4 compared to mice treated with control antibody **(E)**. *p < 0.05, ***p < 0.001. Results were analysed with Student’s t test. (n = 3 lungs in each group). Mice treated with HG4 have a significantly longer survival time when compared to mice treated with isotype control antibody **(F)**. ***p < 0.001. Data were analysed using Mantel–Cox log-rank test; n=14/group.

Intranasal administration of LPS to mice causes a marked recruitment of the inflammatory cells into lung tissues associated with severe lung injury and lung tissue damage. Compared to mice treated with an isotype control antibody, lungs from mice treated with HG4 showed less tissue injury 24h and 48h post LPS administration. Mice treated with isotype control antibody showed a significant lung pathology including higher infiltration of inflammatory cells (attributed mainly to PMNs), thickening of the interstitial membrane, alveolar haemorrhage, and a higher level of oedema compared to mice treated with HG4 antibody (**Figures 7A–D**). The degree of lung injury was evaluated using a semi-quantitative histopathological score to assess the beneficial effect of HG4 in reducing lung pathology during LPS-induced ARDS. The average pathology score was significantly lower in mice treated with HG4 compared to mice receiving an isotype control antibody at all-time points after LPS administration (**Figure 7E**). Lung inflammation and tissue injury associated with LPS administration, when leading to significant ALI and ARDS, required mice to be euthanised. Administration of HG4 significantly improved the survival time of mice after LPS administration compared to mice treated with isotype control antibody (**Figure 7F**).

## Discussion

ARDS and its mild form, acute lung injury (ALI), are fundamental lung pathologies characterised by severe pulmonary inflammation with diffuse damage to the alveolar capillary barrier, resulting in severe hypoxia, poor lung function and high risk of mortality (24). Although the features of ARDS are well defined in humans, not all the complex features of the diseases can be reproduced in mice due to anatomical differences between human and rodent lungs. Inhalation of LPS in mice produces the main criteria that can be used for diagnosis of ARDS in animals. These criteria are tissue injury, alteration of alveolar capillary barrier, induction of an acute inflammatory response, and evidence of physiological dysfunction (23). LPS acts as a potent activator of monocytes/macrophages that release a vast number of inflammatory cytokines such as TNFα, IL1β, IL6, IL8, and TGFβ (25, 26) and has been reported to be present in the BALF of ARDS patients. The response to inhalation of LPS varies between humans and rodents. This difference is mainly due to the presence of pulmonary intravascular macrophages (PIM) in humans that increases the susceptibility to lung injury in humans compared to rodents, so higher doses of LPS are required to induce lung pathology in mice (27). The contribution of complement in LPS-mediated lung injury has been highlighted in many publications (28–32). In 2008, Rittirsch et al. demonstrated that inhibition of C5a does not significantly reduce pulmonary inflammation in a murine model of LPS-mediated lung injury and concluded that complement activation has no role in the pathology (31). This observation may be not conclusive because a high level of C3a can still be produced *via* LP-mediated complement activation, leading to C3a-mediated neutrophil influx. In contrast, others have confirmed that complement activation has a deleterious effect in the lung during LPS exposure. Rabinovici et al. showed that administration of LPS to mice caused microvascular lung injury, characterised by elevated deposition of C3b and C5b-9 on the endothelium of pulmonary vessels and pulmonary oedema associated with recruitment of leukocytes and high protein concentration in the BALF. Pre-treatment of these mice with recombinant human soluble complement receptor-1 (rsCR1) significantly reduced the number of immune cells infiltrating the lung, minimised deposition of C3 and C5b-9 in lung vessels and decreased pulmonary oedema (29). In addition, Kawabata et al. showed that the inhibition of C5 or depletion of complement components using cobra venom factor protected mice from LPS-mediated lung injury and death (28).

Our results clearly demonstrate that LPS activates the LP of complement *via* MBL and CL-11. C3b and C4b were deposited on LPS *via* activation of the LP and the AP, without any involvement of the CP.

Local activation of complement in the lung after LPS administration, with subsequent release of complement activation products C3a and C5a, enhances influx of immune cells into the alveolar space and lung tissues (10). Recruitment of activated neutrophils into the lung *via* the anaphylatoxins C3a and C5a can cause severe damage to the host tissues due to the release of potent reactive oxygen species (ROS) and/or reactive nitrogen species (RNS) (30, 33). Chemotaxis of PMNs into lung is a defining characteristic of ARDS and is considered an indication of overwhelming inflammation in lung tissue (34). Our data clearly show massive infiltration of PMNs into lung tissues after intranasal administration of LPS in mice (**Figures 7A–D**). The number of PMNs infiltrating the lung tissue was significantly reduced when mice were treated with HG4, a derivative of the therapeutic anti-human MASP-2 monoclonal antibody narsoplimab, modified to enhance murine inhibition of MASP-2, inhibiting the LP, compared to mice treated with an isotype control antibody. Moreover, levels of the inflammatory cytokines TNF-a and IL-6 were also significantly lower in mice that received HG4. Several previous publications reported a strong correlation between development of ARDS in patients and high levels of PMNs and inflammatory cytokines in lung tissues (34–38).

Local levels of complement components in lung tissue change during the acute inflammation induced by LPS, presumably through movement of complement proteins from the bloodstream into the alveolar space (30). The LP recognition molecules bind to damaged lung tissue and elicit a secondary complement-mediated inflammatory cascade, which is amplified through the activation of the AP, that further enhances lung tissue damage. This inflammatory reaction is mediated *via* complement activation where L-fucose residues decorating lung epithelial cells specifically bind to CL-11 and elicit LP-mediated complement activation (39–41). Howard et al. recently showed that CL-11 also binds directly to damaged epithelial cells with subsequent activation of the complement system (42). Several studies reported that patients with ARDS show evidence of different degrees of complement activation, the extent of which correlates with the degree and outcome of ARDS (30, 43–45). In this work we clearly demonstrate that LP inhibition, using an antibody against MASP-2, significantly reduces lung inflammation and lung tissue damage after LPS administration and strongly reduces mortality. This finding is supported by significant reductions in (1) the levels of cytokines and LDH in the lung, (2) the concentration of protein in the BALF and (3) the levels of leukocytes and PMNs in the airways and lung tissues.

## Data availability statement

The original contributions presented in the study are included in the article. Further inquiries can be directed to the corresponding authors.

## Ethics statement

Animal procedures were approved by the local Ethical Committee of the Faculty of Pharmacy, Mansoura University, Egypt, or by the University of Cambridge Ethics Committee. Experiments in Cambridge were performed under the Home Office Project Licence numbers PP273509.

## Author contributions

YA, AS, DR, SA-R, PK, and SY designed and performed the experiments. YA, NL, GD, and WS wrote and revised the manuscript. MY, TD, and GD provided essential reagents and revised the manuscript. All authors contributed to the article and approved the submitted version.
